# Induction of Non-Targeted Stress Responses in Mammary Tissues by Heavy Ions

**DOI:** 10.1371/journal.pone.0136307

**Published:** 2015-08-28

**Authors:** Tony J. C. Wang, Cheng-Chia Wu, Yunfei Chai, Roy K. K. Lam, Nobuyuki Hamada, Shizuko Kakinuma, Yukio Uchihori, Peter K. N. Yu, Tom K. Hei

**Affiliations:** 1 Center for Radiological Research, Department of Radiation Oncology, Columbia University, New York, NY, United States of America; 2 National Institute of Radiological Sciences, Chiba, Japan; 3 Department of Physics and Materials Science, City University of Hong Kong, Kowloon, Hong Kong SAR; 4 Radiation Safety Research Center, Nuclear Technology Research Laboratory, Central Research Institute of Electric Power Industry, Tokyo, Japan; National Taiwan University, TAIWAN

## Abstract

**Purpose:**

Side effects related to radiation exposures are based primarily on the assumption that the detrimental effects of radiation occur in directly irradiated cells. However, several studies have reported over the years of radiation-induced non-targeted/ abscopal effects *in vivo* that challenge this paradigm. There is evidence that Cyclooxygenase-2 (COX2) plays an important role in modulating non-targeted effects, including DNA damages *in vitro and mutagenesis in vivo*. While most reports on radiation-induced non-targeted response utilize x-rays, there is little information available for heavy ions.

**Methods and Materials:**

Adult female transgenic *gpt* delta mice were exposed to an equitoxic dose of either carbon or argon particles using the Heavy Ion Medical Accelerator in Chiba (HIMAC) at the National Institute of Radiological Sciences (NIRS) in Japan. The mice were stratified into 4 groups of 5 animals each: Control; animals irradiated under full shielding (Sham-irradiated); animals receiving whole body irradiation (WBIR); and animals receiving partial body irradiation (PBIR) to the lower abdomen with a 1 x 1 cm^2^ field. The doses used in the carbon ion group (4.5 Gy) and in argon particle group (1.5 Gy) have a relative biological effectiveness equivalent to a 5 Gy dose of x-rays. 24 hours after irradiation, breast tissues in and out of the irradiated field were harvested for analysis. Induction of COX2, 8-hydroxydeoxyguanosine (8-OHdG), phosphorylated histone H2AX (γ-H2AX), and apoptosis-related cysteine protease-3 (Caspase-3) antibodies were examined in the four categories of breast tissues using immunohistochemical techniques. Analysis was performed by measuring the intensity of more than 20 individual microscopic fields and comparing the relative fold difference.

**Results:**

In the carbon ion group, the relative fold increase in COX2 expression was 1.01 in sham-irradiated group (p > 0.05), 3.07 in PBIR (p < 0.05) and 2.50 in WBIR (p < 0.05), respectively, when compared with controls. The relative fold increase in 8-OHdG expression was 1.29 in sham-irradiated (p > 0.05), 11.31 in PBIR (p < 0.05) and 11.79 in WBIR (p < 0.05), respectively, when compared with controls. A similar increase in γ-H2AX expression was found in that, compared to controls, the increase was 1.41 fold in sham-irradiated (p > 0.05), 8.41 in PBIR (p < 0.05) and 10.59 in WBIR (p < 0.05). Results for the argon particle therapy group showed a similar magnitude of changes in the various biological endpoints examined. There was no statistical significance observed in Caspase-3 expression among the 4 groups.

**Conclusions:**

Our data show that both carbon and argon ions induced non-targeted, out of field induction of COX2 and DNA damages in breast tissues. These effects may pose new challenges to evaluate the risks associated with radiation exposure and understanding radiation-induced side effects.

## Introduction

Radiation therapy has been essential for the treatment and cure of many malignancies. For many years, a central dogma in radiation biology has been that the effects of radiation occur only in directly irradiated cells and that nuclear DNA is the main target for the biological action of radiation. Accordingly, it has long been presumed that no effect would be expected in cells that receive no radiation; however, this dogma was challenged by the observation of radiation-induced bystander effects [1–3]. This resurgence of interest was stimulated from the findings of Nagasawa and Little who demonstrated that within monolayer cell cultures, a single alpha-particle traversal through <1% of the nuclei leads to the elevated frequency of sister chromatid exchanges in more than 30% of cells [2]. Furthermore, it has been observed that COX2, DNA double-stranded breaks, and oxidative stress appear to play a role in the bystander/ non-targeted response [4–9]. While most data reported on non-targeted effects have been obtained with *in vitro* culture systems, *in vivo* studies are now becoming available. Whole-body X-irradiation of radiosensitive Ptch1+/- mice, with the upper half of the animals shielded, resulted in the induction of medulloblastoma, depicting the non-targeted, out of field response [10]. Tamminga and Kovalchuk reported that cranial irradiation of mice resulted in accumulation of DNA damage in the out of field testis tissue [11]. Most reports on radiation-induced non-targeted response utilize x-rays and there is no data on the effect of heavy ions such as carbon or argon ions. In this study, we address the question of whether exposure of heavy ions *in vivo* to a focal area can generate non-targeted, out-of-field response in breast tissue.

The carbon ions used in this study are used to treat patients at the Heavy Ion Medical Accelerator in Chiba (HIMAC) at the National Institute of Radiological Sciences (NIRS) in Japan. Since 1994, over six thousand patients have been treated with heavy ion radiotherapy using the HIMAC, primarily with carbon ions [12]. Among all types of heavy ions, carbon ions are used for cancer therapy for their particular physical and biological characteristics [13,14]. In terms of their radiobiological effects, carbon ions have about three times the relative biological effectiveness (RBE) and an oxygen enhancement ratio (OER) of about half that of X-rays, allowing less oxygen dependence [13,14]. In addition, carbon and argon ion beams can focus the intensity of the beam at the tumor location while reducing doses to proximal normal tissues. Clinical results have shown that carbon ion radiotherapy has the potential to provide a sufficient radiation dose to the tumor, while having acceptable morbidity in the surrounding normal tissues [15–19]. Treatment with hypofractionation has been successfully carried out for a variety of tumors which can provide treatment for a larger number of patients compared to conventional photons over the same period of time. The effects of heavy ions to induce bystander /non-targeted effects *in vitro* have been reported [20–22]. However, to our knowledge, the study reported here is the first to examine heavy-ion non-targeted effects *in vivo*.

## Methods and Materials

### Animals

**Fig 1:**
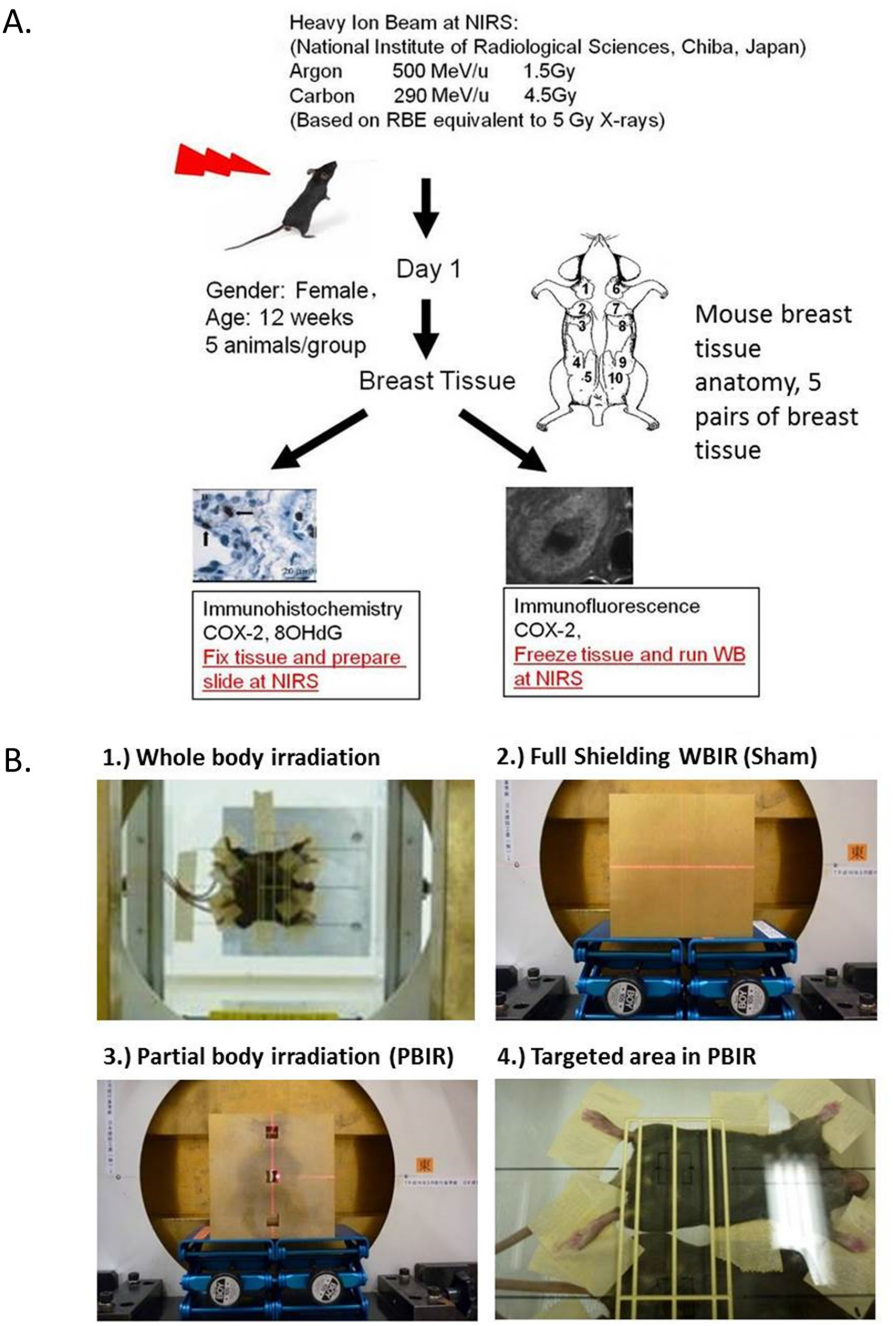
Irradiation set up and experimental design for studies conducted at the NIRS. Adult female transgenic *gpt* mice are exposed to carbon or argon particles and sacrificed 24 hours post-radiation (see text for details). (A) Experimental design. (B) Representative images of shielding techniques for whole body irradiation, sham irradiation, partial body irradiation, and targeted area partial body irradiation.

The Columbia University Institutional Animal Care and Use Committee granted permission for this study. Animal Care Protocol AC-AAAH2850 (Y2 M01) as of 01/06/2015: Approved. The *gpt* delta transgenic mice were obtained from Dr. Takehiko Nohmi of the National Institute of Health Sciences in Japan [23]. The animal facilities and the experiments were approved by the Institutional Animal Care and Use Committee of each respective institutions based on established guidelines. Adult female transgenic *gpt* delta mice, approximately 12 weeks old, we stratified into 4 groups: non-treated control; animals irradiated under full shielding (Sham-irradiated); animals receiving whole body irradiation (WBIR); and animals receiving partial body irradiation (PBIR) to the lower abdomen with a 1 x 1 cm^2^ field (Fig 1). Each group consisted of 5 animals. The Sham-irradiated group served as our secondary control. Each experiment lasted approximately 2 days. Based on our previously published data, the bystander response peaked within 24 hours. Because of this, experiments for Control, Sham-irradiated, and experimental radiation groups were planned for a 24 hour time frame. Anesthetic was provided to each animal with intraperitoneal injection of a combination of ketamine and xylazine (75–95mg/kg and 5 mg/kg, respectively). After set up and irradiation, animals were recovered under a heat lamp until full consciousness was recovered. The mice were then re-examined 24 hours after experiment right before they are sacrificed for tissue harvesting. Terminal dose of ketamine and xylazine was injected. Approximately 15 minutes after injection, pain response was assessed. Once successful analgesic/anesthetic is achieved, cervical dislocation was performed. Such procedure is consistent with the recommendations of the Panel on Euthanasia of the American Veterinary Medical Association.

### Shielding

The block shielding was designed by one of the co-authors Dr. Yukio Uchihori and his colleagues. We considered potential scatter dose even in the face of full shielding as well as partial shielding. To check for possible scatter doses that could confound the result from our out-of-field experiments, we performed measurements of secondary radiation which were mainly lighter ions, including protons and photons. Our measurements, based on the use of GAF films (RTQA-1010, Radiation Products Design, Inc.) and solid-state nuclear-track detectors, showed that the radiation level in the out-of-field breast (mammary glands numbers 1, 2, 3, 6, 7 and 8) was similar to those in the sham-irradiated group and comparable to background radiation level.

### Radiation

We exposed the animals to an equitoxic dose of either 13.1 keV/μm carbon ions (290 MeV/nucleon, 4.5 Gy) or 90.2 keV/μm argon ions (500 MeV/nucleon, 1.5 Gy) accelerated by the HIMAC at the NIRS Chiba, Japan (Fig 1). The doses used in the carbon ion and in argon particle group have an RBE equivalent to a 5 Gy dose of x-rays, a dose which has shown to be effective in the induction of non-targeted response in the *gpt* delta mouse model. The details of the HIMAC beam-delivery system, physical characteristics, biological irradiation procedures, and dosimetry have been described elsewhere [24,25]. 24 hours after radiation, the anterior 3 pairs of mouse breast tissue were harvested for analysis (Fig 1). The posterior 2 pairs of mouse breast tissue were excluded in our analysis because there was concern they may be too close to the irradiation field and thus confound bystander effects.

### Immunohistochemistry

Twenty four hours post-irradiation, animals were euthanized and breast tissues were excised and fixed in buffered formalin. The mammary tissues were embedded in paraffin, and cut in thin histological sections. These sections were transported back to Columbia University and deparaffinated and rehydrated according to standard procedures. For antigen retrieval, heated citrate buffer was used. The slides were treated with 0.3% H_2_O_2_ to suppress endogenous peroxidase activity and subsequently washed with PBS solution. The slides were incubated overnight with a primary monoclonal antibody with either COX2 (Cayman Chemical, Ann Arbor, Michigan), 8-OHdG (Abcam, Cambridge, Massachusetts), γ-H2AX (Cell Signaling Technology, Danvers, Massachusetts), or Caspase-3 (Cell Signaling Technology, Danvers, Massachusetts) antibody. The sections were then incubated in chronological order with a biotinylated secondary antibody for 30 minutes, ABC-reagins for 30 minutes and peroxidase substrate for development for 2 to 10 minutes depending on the intensity. Between incubations, the sections were washed for 10 minutes in PBS solution. The immunoreactivity was determined in at least 50 randomly chosen cells. Negative controls were obtained by omission of the primary antibody on one slide and the secondary antibody in the other. Immunohistochemistry (IHC) staining was analyzed using an Olympus CX31 microscope connected with a Motic MC Camera (2.0 megapixel; MC2001interface, Sterling Heights, MI, USA). Protein expression was measured using Paint Shop Pro (Mountain View, CA, USA). Selected sample areas with no cells involved were measured by setting an arbitrary threshold of 125 (background nonspecific staining level). This area was then used to determine the relative staining in the stained region.

### Statistical Analysis

For statistical analysis of relations between COX2, γ-H2AX, and 8-OHdG to the different radiation treatment arms, the two-tailed t-test was used. A p-value less than 0.05 was considered significant. Statistical analysis was performed using the Windows Excel program.

## Results

**Fig 2:**
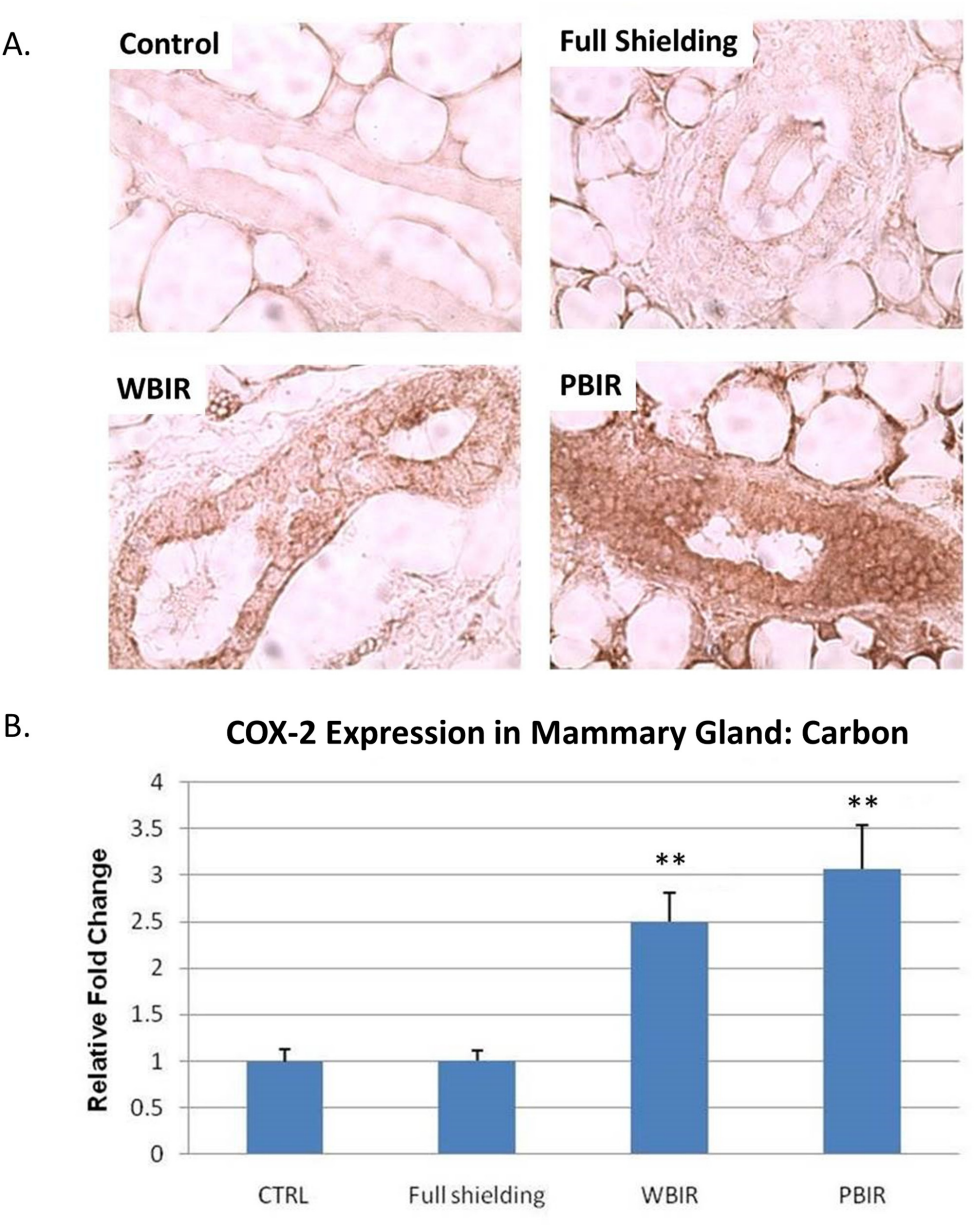
Immunohistochemical staining for COX2 in adult female transgenic *gpt* mice breast epithelial cells in the four categories treated with carbon ion radiotherapy. (A) Representative stains for each of the four categories. (B) Relative fold changes (**p<0.05).

**Fig 3:**
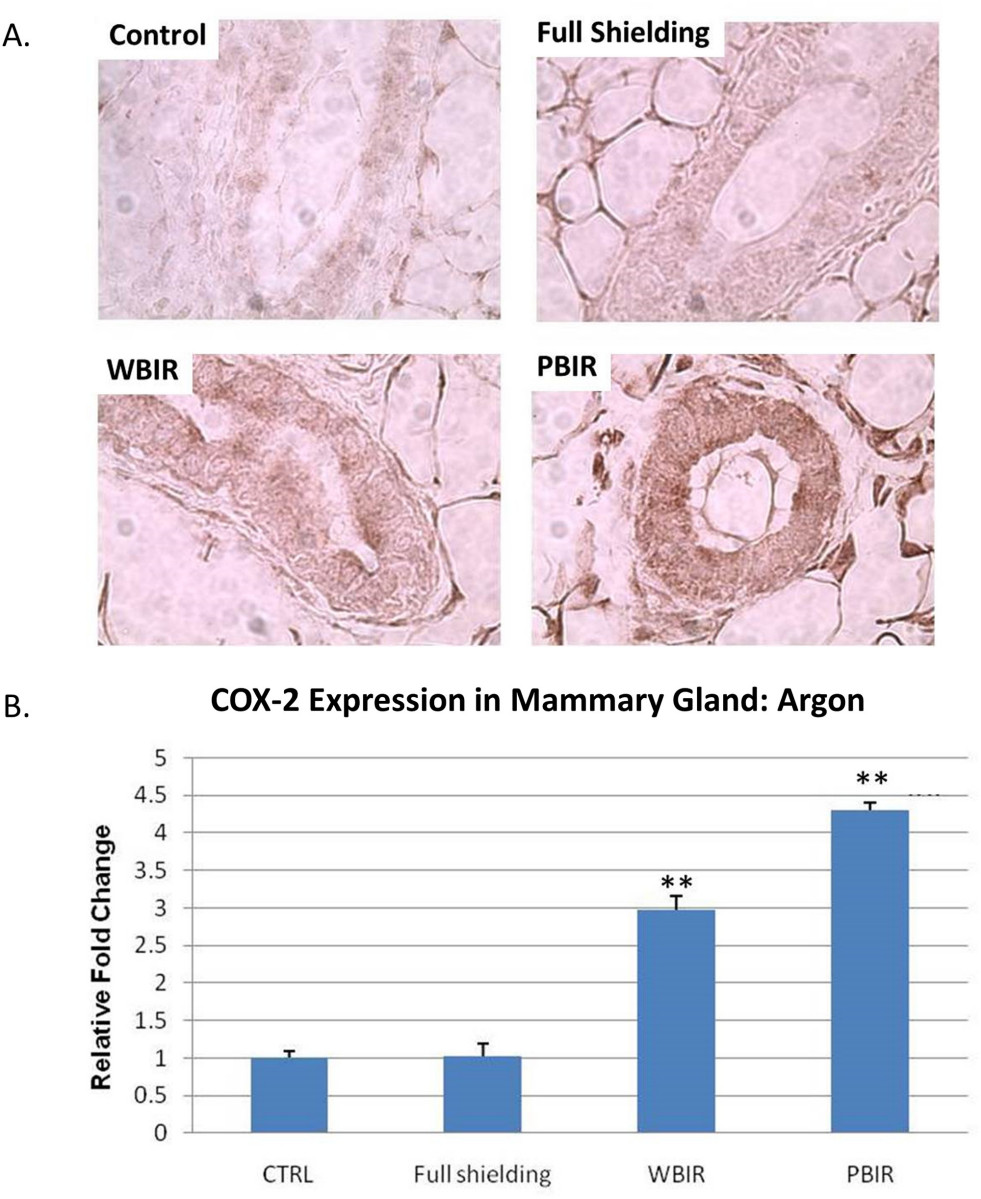
Immunohistochemical staining for COX2 in adult female transgenic *gpt* mice breast epithelial cells in the four categories treated with argon ion radiotherapy. Representative stains for each of the four categories. (B) Relative fold changes (**p<0.05).

Expression of COX2 was examined in the four categories of breast tissues using IHC staining. In the carbon ion treatment groups, the relative fold increase (RFI) in COX2 expression was 1.01 in the sham-irradiated group (p > 0.05), 3.07 in PBIR (p < 0.05) and 2.50 in WBIR (p < 0.05) groups, respectively, when compared with controls (Fig 2). In the argon ion treatment groups, the RFI in COX2 expression was 1.02 in the sham-irradiated group (p > 0.05), 4.30 in PBIR (p < 0.05) and 2.97 in WBIR (p < 0.05) groups, respectively, when compared with controls (Fig 3). The results are similar between both carbon and argon treated groups.

**Fig 4:**
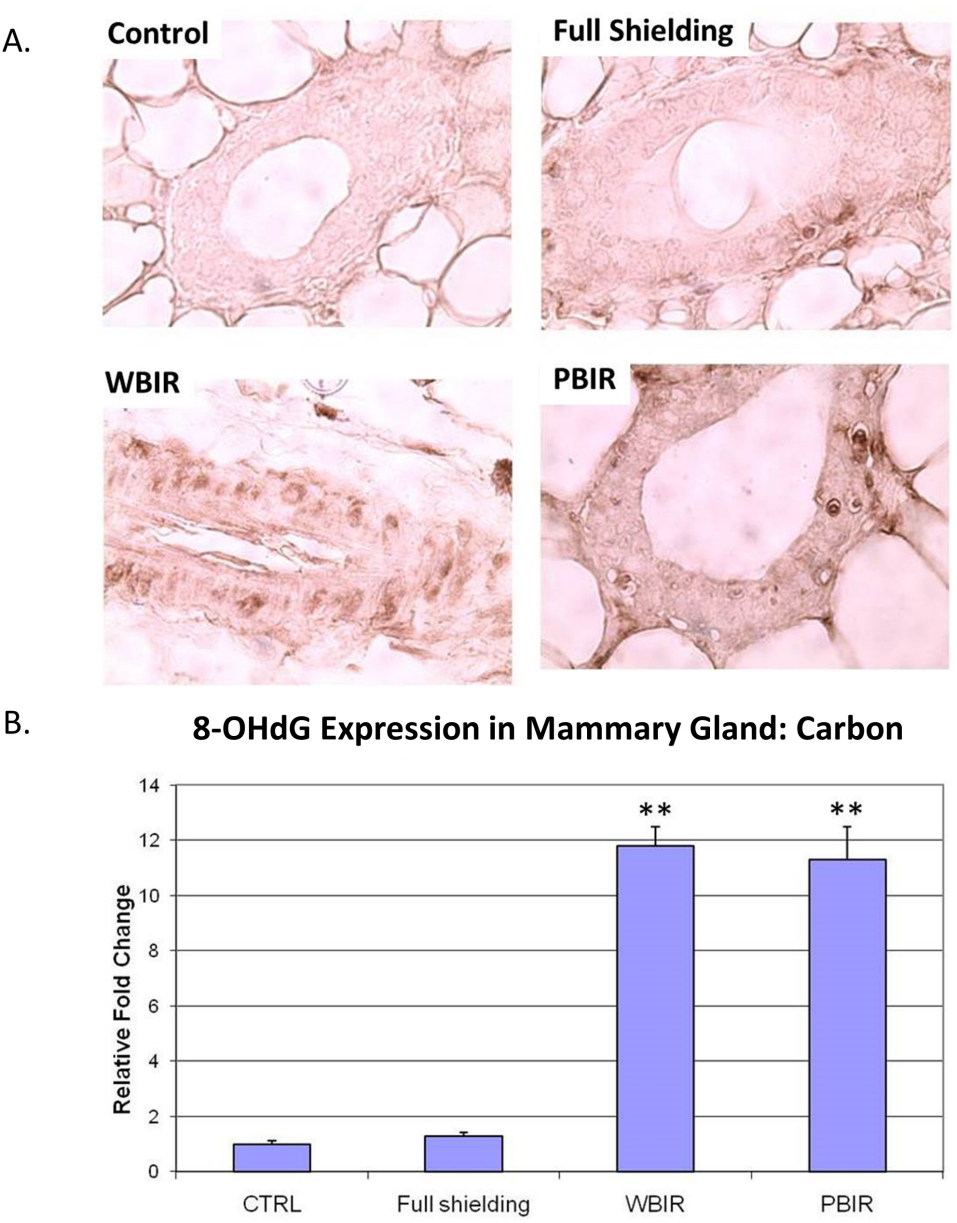
Immunohistochemical staining for 8-OHdG in adult female transgenic *gpt* mice breast epithelial cells in the four categories treated with carbon ion radiotherapy. Representative stains for each of the four categories. (B) Relative fold changes (**p<0.05).

**Fig 5:**
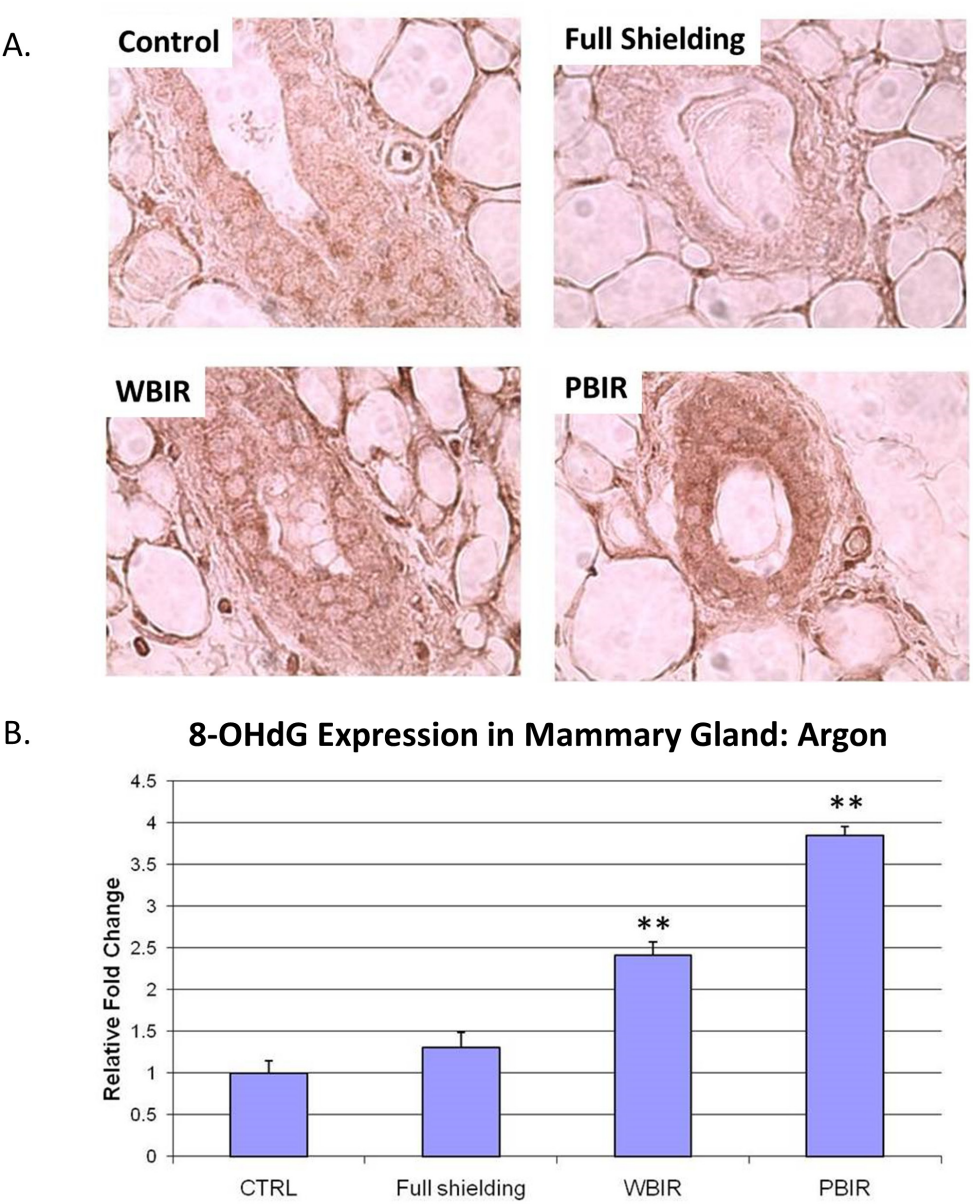
Immunohistochemical staining for 8-OHdG in adult female transgenic *gpt* mice breast epithelial cells in the four categories treated with argon ion radiotherapy. Representative stains for each of the four categories. (B) Relative fold changes (**p<0.05).

Expression of 8-OHdG was examined in the four categories of breast tissues using IHC techniques. In the carbon ion treatment groups, the RFI in 8-OHdG expression was 1.29 in the sham-irradiated group (p > 0.05), 11.31 in PBIR (p < 0.05) and 11.79 in WBIR (p < 0.05) groups, respectively, when compared with controls (Fig 4). In the argon ion treatment groups, the RFI of 8-OHdG expression as 1.30 in the sham-irradiated group (p > 0.05), 3.85 in PBIR (p < 0.05) and 2.41 in WBIR (p < 0.05) groups, respectively, when compared with controls (Fig 5). The results are similar between both carbon and argon treated groups.

**Fig 6:**
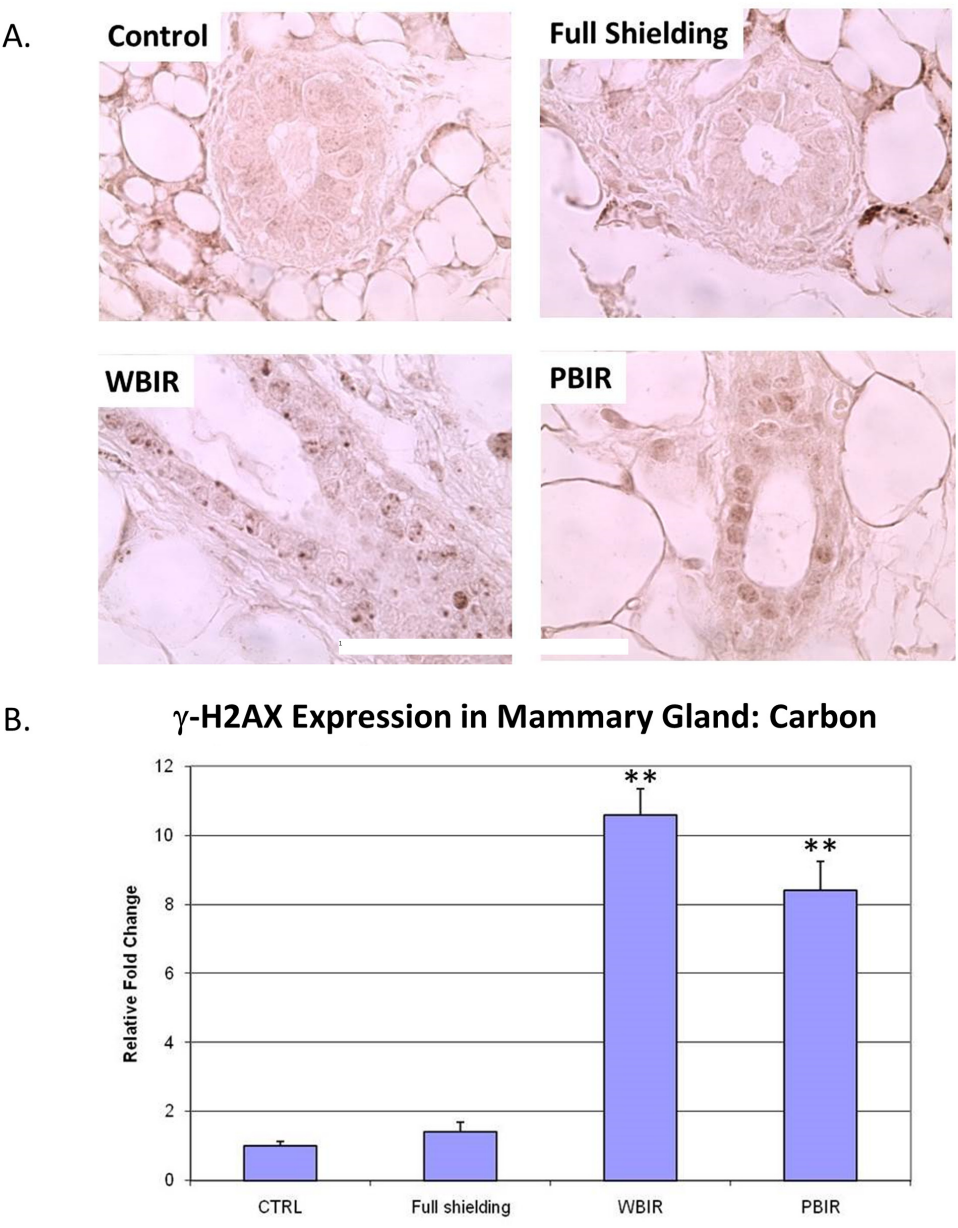
Immunohistochemical staining for H2AX in adult female transgenic *gpt* mice breast epithelial cells in the four categories treated with carbon ion radiotherapy. Representative stains for each of the four categories. (B) Relative fold changes (**p<0.05).

**Fig 7:**
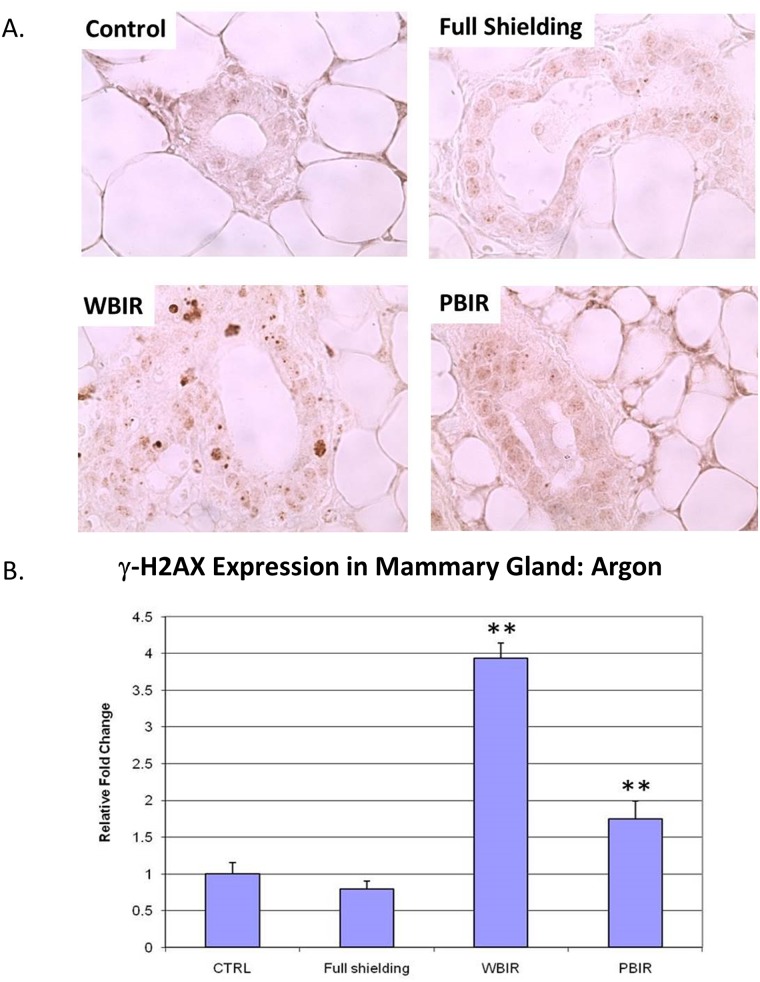
Immunohistochemical staining for H2AX in adult female transgenic *gpt* mice breast epithelial cells in the four categories treated with argon ion radiotherapy. Representative stains for each of the four categories. (B) Relative fold changes (**p<0.05).

**Fig 8:**
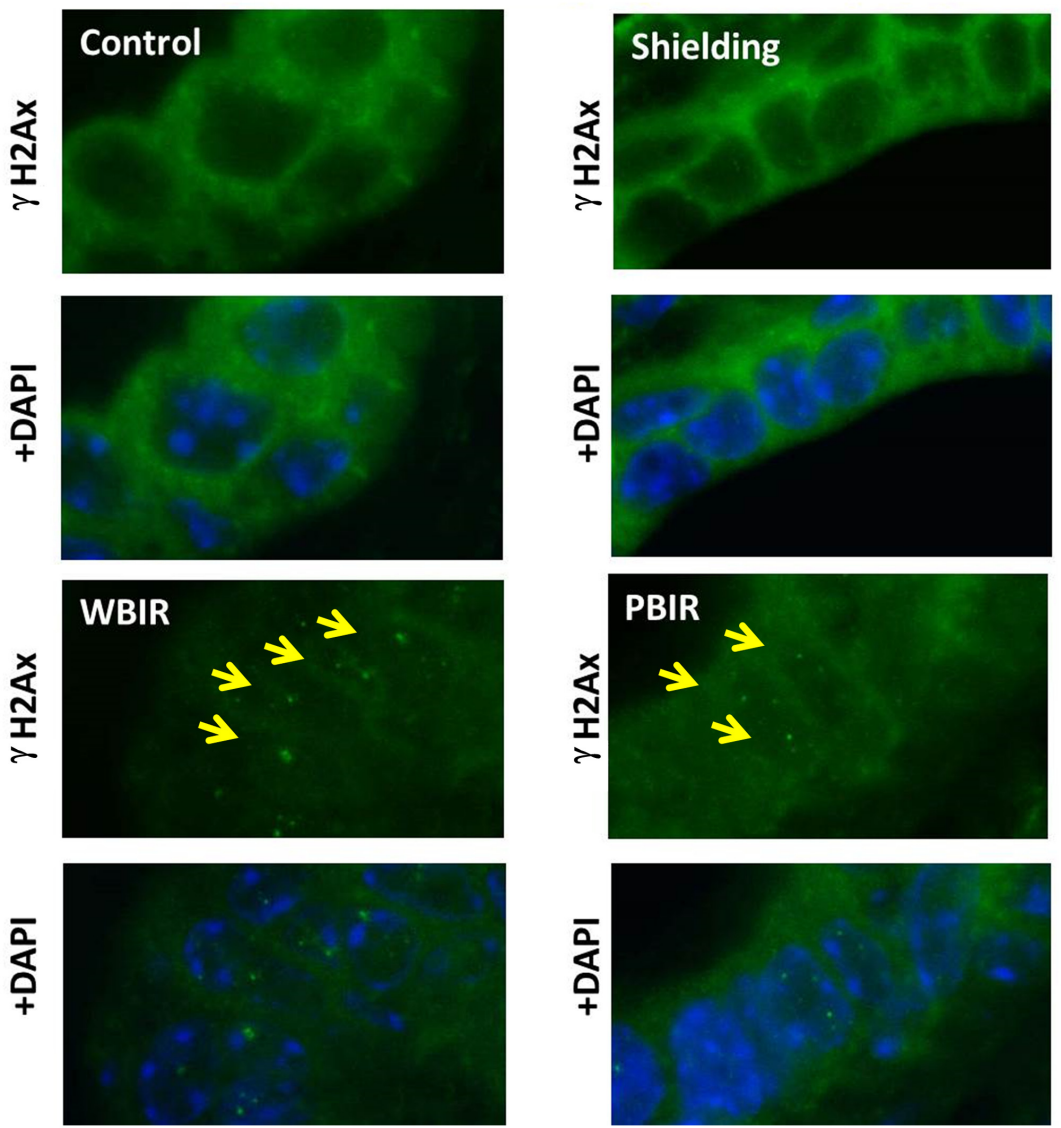
Immunofluorescence staining for γ-H2AX (green) in adult female transgenic *gpt* mice breast epithelial cells in the four categories treated with carbon ion radiotherapy. Slides were co-stained with DAPI (blue). Yellow arrows identify γ-H2Ax foci.

Expression of γ-H2AX was examined in the four categories of breast tissues using IHC techniques. Analysis was performed by comparing the foci number, foci size, and foci intensity in different categories. In the carbon ion group, the RFI in γ-H2AX expression was 1.41 in the sham-irradiated group (p > 0.05), 8.41 in PBIR (p < 0.05) and 10.59 in WBIR (p < 0.05) groups, respectively, when compared with controls (Fig 6). In the argon particle therapy group, the RFI of γ-H2AX expression was 0.80 in the sham-irradiated group (p > 0.05), 1.75 in PBIR (p < 0.05) and 3.93 in WBIR (p < 0.05) groups, respectively, when compared with controls (Fig 7). The data are consistent with the results obtained using immunofluorescent staining in the carbon ion group, where the number of γ-H2AX foci per nucleus was 0.075 in the control group, 0.059 in the sham-irradiated group (p > 0.05), 0.224 in WBIR group (p < 0.05), and 0.153 in PBIR group (p < 0.05) (Fig 8).

There was no statistical significance observed in the Caspase-3 expression among the 4 groups in either carbon or argon particle therapy 24 hours after exposure (S1 and S2 Figs).

## Discussion

A major paradigm shift in radiation biology in the last decade has resulted from the elucidation of the biological consequence of targeted cytoplasmic irradiation and discovery of the non-targeted effect. Radiation-induced non-targeted effects have been well documented in *in vitro* and *in vivo* studies.

Mechanism-based studies can provide insight on the nature of the non-targeted signaling process and in assessing the clinical relevance of the phenomena. From animal studies with X-rays, there is evidence that partial irradiation of the lungs can induce a non-targeted response in the non-irradiated part of the lungs through the induction of inflammatory cytokines [26]. With irradiation of the lower region of the lungs, the frequency of micronuclei increased in the out-of-field upper lungs relative to the sham-irradiated group. The induction of micronuclei in the non-targeted lung tissues was inhibited by superoxide dismutase (SOD) and L-N^G^-Nitroarginine methyl ester (L-NAME), a non-specific inhibitor of nitric oxide synthase, which suggested that production of reactive oxygen species and nitric oxide resulted in indirect DNA damage and induced a bystander effect in the neighboring tissue [27,28]. Furthermore, our recent studies show that irradiation of the lower abdomen of mice with X-rays results in the induction of an inflammatory response as well as mutations, and COX2 induction in out-of-field lung tissues. Furthermore, there is recent evidence that the transforming growth factor beta and the transforming growth factor beta receptors mediated COX2 signaling pathway play a critical role in radiation non-targeted response [29,30]. Finally, using the radiosensitive *Patched-1* ^+/-^ (*Ptch1* ^+/-^) mouse model system that has a defect in radiation-induced activation of the ATR-Chk1 checkpoint signaling pathway, Mancuso *et al*. reported induction of medulloblastoma in the non-irradiated brain tissues after partial irradiation of the lower half of the animal with a 3 Gy dose of X-rays[10]. Thus, while many studies on bystander effects have been performed with low LET X-rays, data on heavy-ion radiation-induced non-targeted response *in vivo* remain limited.

In the past decade, heavy ion radiotherapy has shown promising results with acceptable morbidity in adjacent normal tissues for cancer patients [15–19]. With longer patient survival, potential late sequelae such as bystander effects are becoming a matter of great interest. Bystander effects may be of importance in secondary cancer induction, which has been shown to increase with improved primary tumor cure rates from conventional photons [31]. Understanding the mechanism of bystander effects is important and may produce novel ways to enhance existing targeted radiotherapy approaches and/or reduction of secondary cancer risk. For example, Zhou et al. was able to significantly reduce bystander effects by suppressing COX2 activity [9].

There are some studies of heavy ion-induced bystander effects *in vitro*. Harada et al. was able to show bystander killing of human lung cancer cell lines using carbon ion radiation therapy [32]. Furthermore, Hamada et al. was able to inactivate clonogenic potential of fibroblast bystander cells using carbon ions [33]. In a recent study, Ponnaiya et al. was able to show that heavy ions can induce bystander effects and that both irradiated and bystander cells can exhibit chromosomal instability [34]. To our knowledge, there is sparse data for bystander effects *in vivo*.

Our study was to investigate whether heavy ion radiation *in vivo* can induce bystander effects on breast tissue. We chose markers that play a role in x-ray bystander effects such as COX2, 8-OHdG, and γ-H2AX. We treated the animals with a setup to simulate a clinical scenario. In our prior experience with photon based bystander effect in gpt delta transgenic mice in vivo, we demonstrated using kinetic studies that COX2 expression in the lungs of mice treated with WBIR (whole body irradiation) peaked after 24-hours in both male and female mice by western blot staining [29]. Using this time point, shielding studies that examined bystander effect was performed. gpt delta transgenic mice that received WBIR or PBIR (partial body irradiation) both showed increased COX2 IHC staining of the lung bronchial epithelial cells at 24-hours after irradiation. Given that the peak COX2 expression pattern occurred at 24-hours, down-stream effectors of COX2 expression were examined at 24-hours including prostaglandins PGE2 and PGF2, in which both were elevated in the PBIR group at 24-hours. In addition, 8-OHdG, a marker for oxidative DNA damage, was elevated in the PBIR and WBIR groups at 24-hours. Since nothing is known about the effects of Heavy-ion and bystander effect, we elected to select a well establish model for bystander effect by using the gpt delta transgenic mice model. Furthermore, we elected to use the 24-hour time point to examine expression pattern of 8-OHdG and γ-H2AX to assess for potential downstream effects of COX2 over-expression. Our results show that heavy ion radiation can induce bystander effects in breast tissue similarly to x-rays. In our previous experience with photon based bystander effect in the lungs, increased COX2 and 8-OHdG expression were associated with increased Caspase-3 staining [29]. However, in the heavy-ion model in breast tissue, this correlation was not observed. Possible causes for this discrepancy could be that the oxidative stress from the bystander effect activated alternative pathways including p53 induced protein containing death domain (PIDD) to Caspase-2 mediated apoptosis pathway as opposed to Caspase 3 mediated apoptosis [35]. Further investigation is needed. The fact that similar markers including COX2, 8-OHdG, and γ-H2AX are upregulated in a similar fashion suggests that the mechanism for heavy ion bystander effects may be similar to x-ray bystander effects; however, differences in Caspase-3 staining suggest possible differences.

Interestingly, the staining patterns for γ-H2AX in the WBIR (whole body irradiation) group differ from that of the PBIR (partial body irradiation) PBIR group. γ-H2AX staining is more diffused in the breast tissue from mice receiving PBIR as opposed to distinct foci seen in the WBIP group. H2AX proteins play an important role in DNA repair. Upon DNA double-strand break (DSB), HS2AX proteins are rapidly phosphorylated on a serine four residues from the carboxyl terminus to form γ-H2AX. γ-H2AX form large numbers of molecules in the chromatin around the break site creating foci that are visible on immunohistological staining. In addition to forming foci from DSB, the formation of γ-H2AX can occur through alternative pathways. These non-DSB mechanisms can sometimes result in a diffuse pattern of γ-H2AX staining, also known as pan-nuclear staining [36]. The speculated staining pattern of γ-H2AX in the WBIR (Fig 6A) is consistent with whole body irradiation as gamma irradiation leads to DNA double strand breaks. Increased staining for γ-H2AX occurred in a pan-nuclear staining pattern in tissue from mice treated with PBIR (Fig 6A). Pan-nuclear staining of γ-H2AX has been associated with ultraviolet C radiation through ATR kinase activity and TNF-related apoptosis-inducing ligand (TRAIL)-induced apoptosis [37,38]. Interestingly, TRAIL signaling has been associated with radiation induced bystander effect [39]. Further investigation is needed to identify whether this is the case.

In summary, our data show that heavy ions can induce bystander effects in out-of-field breast tissues with upregulation of COX2, 8-OHdG, and γ-H2AX. These effects may pose new challenges to evaluate the risks associated with radiation exposure and understanding radiation-induced side effects. Therefore, continuing to investigate the consequences of heavy ion radiation-induced bystander effects may lead to better understanding of both acute and late-side effects in a clinical setting.

## Supporting Information

S1 Fig Immunohistochemistry staining for Caspase-3 in adult female transgenic *gpt* mice breast epithelial cells in the four categories treated with carbon ion radiotherapy. Representative stains for each of the four categories.
(TIF)

S2 Fig Immunohistochemistry staining for Caspase-3 in adult female transgenic *gpt* mice breast epithelial cells in the four categories treated with argon ion radiotherapy. Representative stains for each of the four categories.
(TIF)
