# Supplementary figures and images for: Induction of Non-Targeted Stress Responses in Mammary Tissues by Heavy Ions
Source: PLoS One. 2015 Aug 28;10(8):e0136307. doi: 10.1371/journal.pone.0136307 (PMC4552651; doi:10.1371/journal.pone.0136307)

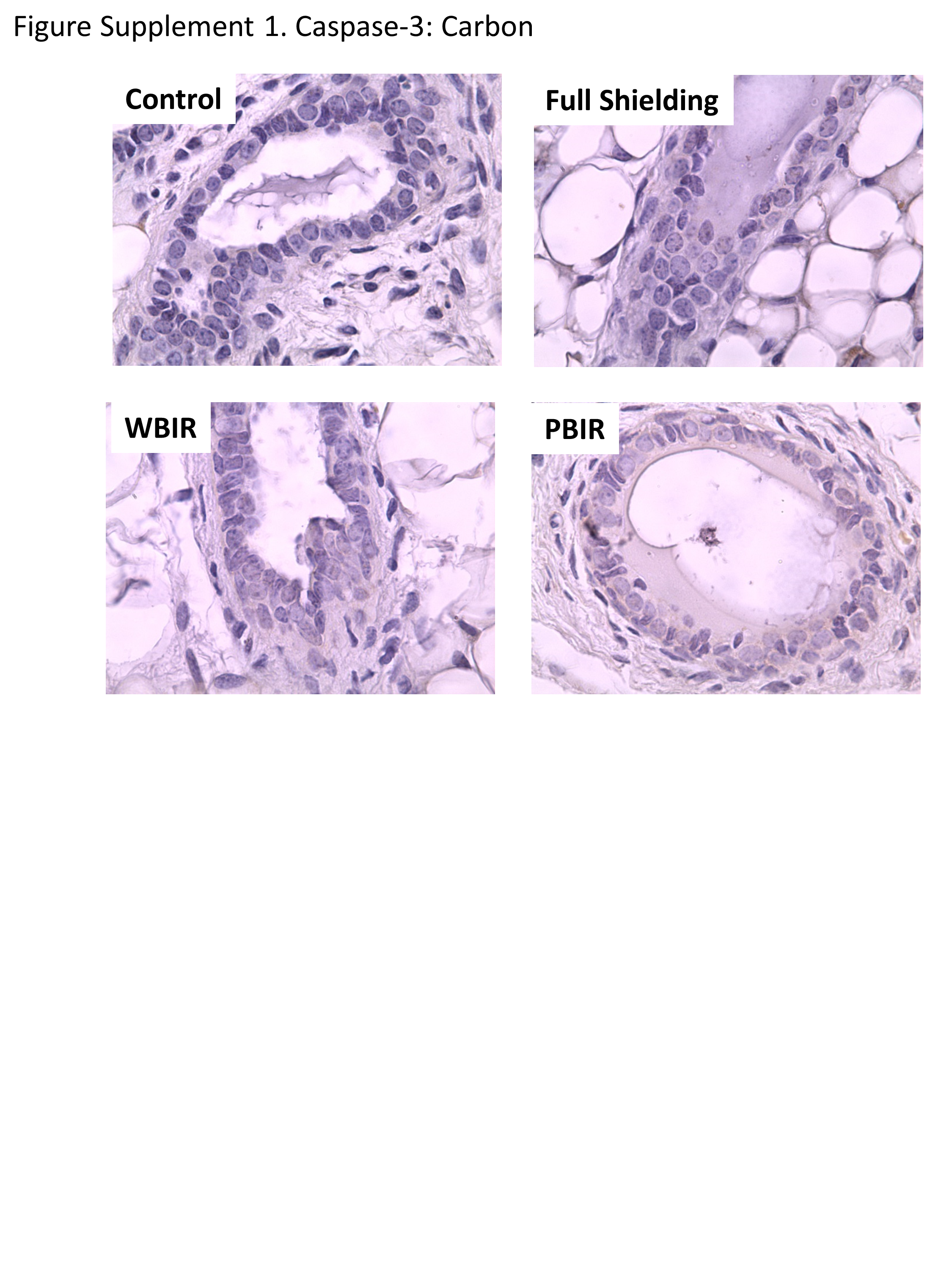

Supplement: S1 Fig — Representative stains for each of the four categories. (TIF) [file pone.0136307.s001.tif]

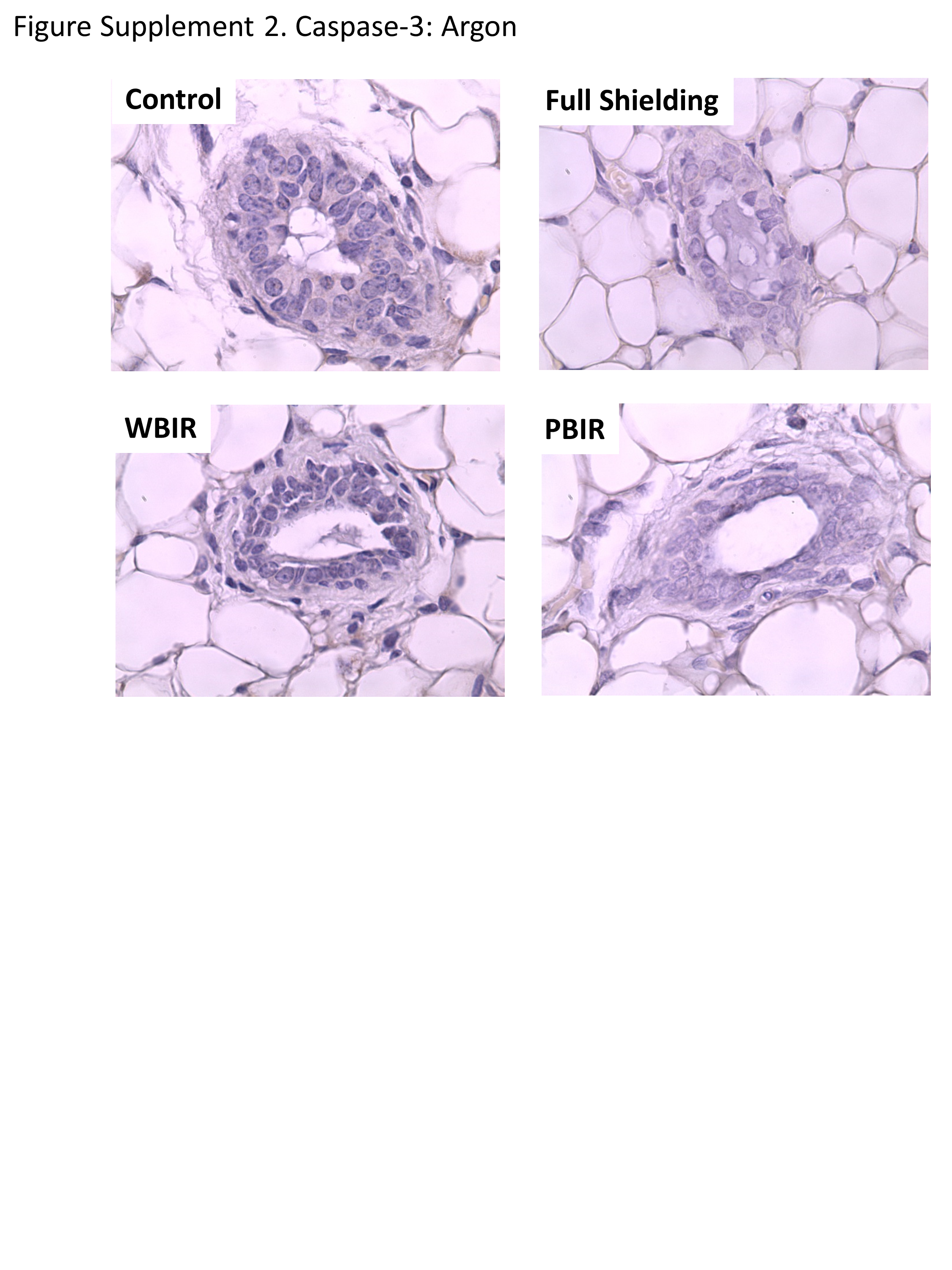

Supplement: S2 Fig — Representative stains for each of the four categories. (TIF) [file pone.0136307.s002.tif]
